# Comparative genomics and genotype-phenotype associations in *Bifidobacterium breve*

**DOI:** 10.1038/s41598-018-28919-4

**Published:** 2018-07-13

**Authors:** Francesca Bottacini, Ruth Morrissey, Maria Esteban-Torres, Kieran James, Justin van Breen, Evgenia Dikareva, Muireann Egan, Jolanda Lambert, Kees van Limpt, Jan Knol, Mary O’Connell Motherway, Douwe van Sinderen

**Affiliations:** 1 APC Microbiome Ireland, University College Cork, Western Road, Cork, Ireland; 2 School of Microbiology, University College Cork, Western Road, Cork, Ireland; 3 Danone Nutricia Research, Utrecht, The Netherlands; 4 Laboratory of Microbiology, Wageningen University, Wageningen, The Netherlands

## Abstract

Bifidobacteria are common members of the gastro-intestinal microbiota of a broad range of animal hosts. Their successful adaptation to this particular niche is linked to their saccharolytic metabolism, which is supported by a wide range of glycosyl hydrolases. In the current study a large-scale gene-trait matching (GTM) effort was performed to explore glycan degradation capabilities in *B*. *breve*. By correlating the presence/absence of genes and associated genomic clusters with growth/no-growth patterns across a dataset of 20 *Bifidobacterium breve* strains and nearly 80 different potential growth substrates, we not only validated the approach for a number of previously characterized carbohydrate utilization clusters, but we were also able to discover novel genetic clusters linked to the metabolism of salicin and sucrose. Using GTM, genetic associations were also established for antibiotic resistance and exopolysaccharide production, thereby identifying (novel) bifidobacterial antibiotic resistance markers and showing that the GTM approach is applicable to a variety of phenotypes. Overall, the GTM findings clearly expand our knowledge on members of the *B*. *breve* species, in particular how their variable genetic features can be linked to specific phenotypes.

## Introduction

Bifidobacteria are commonly encountered, Gram-positive, rod-shaped, anaerobic and saccharolytic commensals of the gastrointestinal tract of mammals, including humans, where their presence is believed to contribute to the maintenance of a healthy gut^1,2^. The positive effects that have been attributed to bifidobacteria include reinforcement of the host intestinal barrier, competitive exclusion of pathogens, modulation of the immune response, (micro)nutrient supplementation, and enhancement/expansion of host metabolism^3–5^. It has been shown that the microbiota of healthy newborns is highly populated by ‘infant type’ bifidobacteria, such as *Bifidobacterium breve*, *Bifidobacterium longum* spp. *longum*/*infantis* and *Bifidobacterium bifidum*^6,7^. In this context, members of these species are believed to play a crucial role in the healthy development of the infant gut^8–10^.

Bifidobacteria rely on diet- and/or host-derived glycans (such as human milk oligosaccharides in the case of ‘infant type’ bifidobacteria) to support their metabolic activities and persistence in the gut^11–13^. For this reason the *Bifidobacterium* pan-genome consists of a relatively high percentage (~13.5%) genes assigned to glycan metabolism^14^ and their glycan-degrading capabilities can be further expanded through resource-sharing and cross-feeding strategies involving other members of the gut community^15–17^.

*B*. *breve*, a common bifidobacterial member of the infant microbiota^6,18,19^, represents one of the most extensively studied and characterized bifidobacterial species from a comparative and functional genomics perspective. A prototypical strain of this species, *B*. *breve* UCC2003, has been the subject of a considerable number of functional studies, revealing this strain’s ability to utilize host-derived glycans (e.g. various human milk oligosaccharides, and mucin-derived sulphated sugars, fucose and sialic acid)^16,20,21^, as well as a range of dietary, mostly plant-derived, mono-, oligo- and polysaccharides (such as glucose, fructose, ribose, sucrose, lactose, melezitose, raffinose, cellodextrin, galactan and starch)^21–28^.

Based on a comparative and pan-genome analysis conducted on thirteen representatives of the *B*. *breve* species, variable genetic features (the variome) were identified that are responsible for strain diversification, including genes involved in host or environment interactions, such as biosynthesis of cell surface-exposed structures and exopolysaccharide (EPS), and in defense mechanisms active against invading foreign DNA (i.e. CRISPR/Cas systems and R/M systems)^22^. In addition, these *B*. *breve* representatives exhibit a diverse range of glycan hydrolytic activities, which are typically encoded by carbohydrate-specific utilization clusters that commonly also contain genes specifying a transcriptional regulator and components of ABC transport systems^29^.

A combination of *in silico* comparative analysis and experimental data on growth on a number of different carbon sources has shown that a so-called gene-trait matching (GTM) approach can be employed to elucidate genes responsible for carbohydrate metabolism by *B*. *breve*^22^. The current study represents a substantial expansion of our previous phenotypic investigation of this species. By applying GTM to a dataset of 20 *B*. *breve* genomes tested for their growth performance on 77 different saccharidic substrates, we identified two previously uncharacterized carbohydrate utilization gene clusters. Furthermore, extension of our approach to EPS production and antibiotic resistance resulted in novel findings which significantly expands our knowledge on members of this bifidobacterial species.

## Results and Discussion

### Comparative genomics of 20 *B*. *breve* strains and relative pan-genome

In the present study a comparative genome analysis of 20 genetically distinct strains of *B*. *breve* was carried out to serve as a basis for a comprehensive GTM analysis, aimed at linking genetic features with strain-specific phenotypic properties. The genome sequences of the selected isolates had previously been obtained as part of a comparative methylome analysis of *B*. *breve* aimed at increasing the genetic accessibility of member of this species^30^. From this strain collection we selected a subset of 20 non-clonal *B*. *breve* representatives (Supplemental Table S1), for each of which Pacbio genome sequencing and subsequent assembly resulted in a single genome contig with over 100-fold sequence coverage. These strains were subjected to a comprehensive phenotypic assessment, which also included strain *B*. *breve* UCC2003 as a prototype for this species^31^.

**Figure 1:**
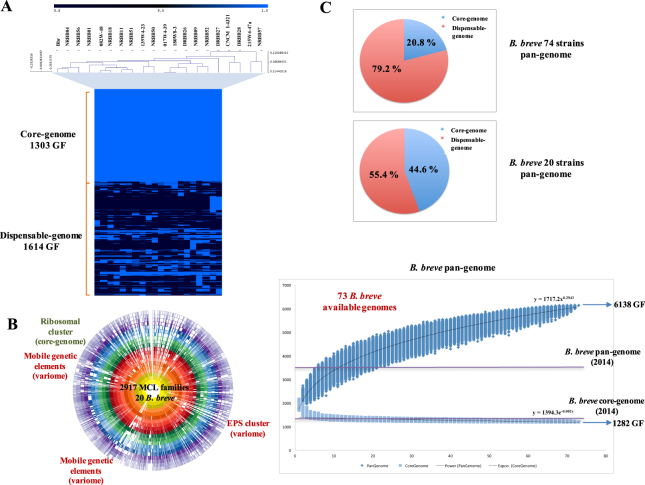
Comparative genomics of 20 *Bifidobacterium breve* strains. Representation of comparative genomics and pan-genome analysis conducted on 20 *B*. *breve* strains and additionally compared to 53 publicly available *B*. *breve* representatives. Panel A: Heatmap representing two-way hierarchical clustering analysis (HCL) conducted on the 20 *B*. *breve* genomes. Estimation of core-genome and dispensable-genome is also indicated in gene families (GF). Panel B: Circular plot representing the distribution of MCL families among *B*. *breve* strains relative to their position along the chromosome. Absence of families are indicated by white regions, while elements of core-genome and dispensable-genome are also highlighted. Panel C: Pan-genome analysis of 73 *B*. *breve* representatives, consisting of the 20 strains used in this study as well as 53 additional, publicly available genomes. Pan-genome and core-genome sizes are indicated, as well as the previously estimated pan-genome size (Bottacini *et al*., 2014) for comparative purposes.

In order to provide an updated and comprehensive estimation of the *B*. *breve* pan-genome and to determine which fraction of the total gene content assigned to this species is represented by the 20 assessed strains, a pan-genome analysis was performed among 73 *B*. *breve* representatives available in public databases inclusive of the 20 strains which were selected for GTM analysis within this study (Supplemental Table S2). Strains were selected for pan-genome analysis based on the degree of completeness of their genome sequence (total number of bases > 2 Mbp). Comparing our current analysis with a previous report^22^, we observed that the core-genome previously computed for 13 *B*. *breve* representatives (1307 gene families) is only slightly larger in size than the one obtained from 73 representatives (1282 gene families). This indicates that the analysis conducted on 13 representatives already provided a comprehensive overview of the core structure of the genome of this species. In contrast, the size of the calculated pan-genome nearly doubled (total number of 6138 gene families *vs* the previously computed 3667) (Fig. 1). In the present study the inclusion of 73 representatives in the pan-genome calculation (curve determined as least squares fit of the power law *n = k N*^*γ*^)^32^ resulted in an exponent γ = 0.29 consistent with an open pan genome (0 < γ < 1), although it appears to be approaching saturation (γ = 0). A closer inspection of the dataset revealed that 69% of the variome in *B*. *breve* consists of Truly Unique Genes (TUGs), which represent > 99.9% of the new genes discovered in the pan-genome after the 37^th^ iteration. The predicted functions of these TUGs indicate that at least 50% of these genes encode hypothetical, uncharacterized proteins, or genes associated with mobile genetic elements. An analysis of the relative G + C mol% revealed that 47% of the identified TUGs possess a G + C content that deviates from the average (59% ± 4%), suggesting that horizontal gene transfer is an important contributor to the acquisition of new genes within the species. It is worth mentioning that horizontal DNA transfer by conjugation has previously been described in bifidobacteria^33,34^. This also suggests that such DNA transfer is a factor which will continue to impact on pan-genome determinations of *B*. *breve*, particularly if other bifidobacterial groups involved in this genetic exchange share the same environment.

Comparative analysis of the 20 *B*. *breve* strains selected for GTM analysis showed that 1303 gene families occur in all examined strains (thus constituting the core-genome of this group), while the remaining 1614 gene families are present in some, but not all, members (thus representing the dispensable genome of these 20 strains). Of the 1614 gene families that make up the dispensable genome, 385 occur in just one representative (constituting the TUGs of this *B*. *breve* group) (Fig. 1). Therefore, based on our knowledge of this species to date, the dataset of 20 *B*. *breve* strains selected for the determination of GTM associations represents approximately 33% of the known dispensable genome of *B*. *breve* (Fig. 1).

### The *B*. *breve* glycobiome and associated growth profiles

**Figure 2:**
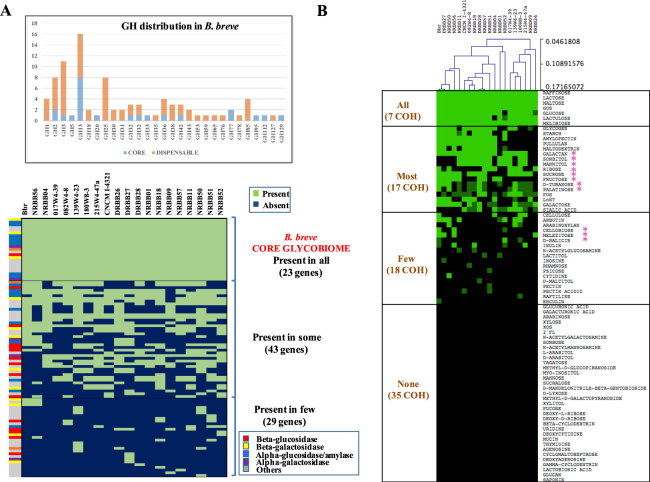
The *B*. *breve* glycobiome and fermentation profiles. Representation of the distribution of glycosyl hydrolases (GHs) across *B*. *breve* and carbon source utilization. Panel A: Predicted GH-encoding gene content of 20 *B*. *breve* genomes displayed as a presence/absence heatmap. An additional bar chart indicates the abundance of each GH family across the 20 *B*. *breve* strains. Panel B: Heatmap representing the fermentation profiles of the 20 *B*. *breve* strains as based on 77 different substrates.

The *in silico* prediction of the glycosyl hydrolase (GH) content encoded by the 20 *B*. *breve* genomes, designated here as the *B*. *breve* glycobiome, identified 95 orthologous genes organized in 29 GH families. Based on our BLASTP and Cazy-mediated GH profiling efforts (see methods section), *B*. *breve* appears to possess a consistent number of enzymes active towards α-glucosidic linkages (18 orthologues belonging to families GH13 and GH31) usually present in di-, oligo-and poly-saccharides (e.g. maltose, starch and related α-glucans) (Fig. 2A). Of note, the GH13 family is represented by various paralogs within a given *B*. *breve* genome^35,36^. The apparent importance of *B*. *breve*’s ability to hydrolyse α-glucosidic linkages is supported by the finding that among the 23 GH-encoding gene families being present in all 20 analysed strains (*B*. *breve* core-glycobiome; Fig. 2A) eight belong to GH13. Considering that *B*. *breve* is particularly abundant in early life where starch-containing foods are among the first digestible dietary carbohydrates introduced at weaning^37^, the observed abundance of GH13 in this species may reflect the importance of hydrolysing α-glucosidic linkages for their colonization and persistence in the (infant) gut.

The second most frequently identified group of carbohydrate-active enzymes in *B*. *breve* is represented by the β-glucosidases (belonging to the GH1 and GH3 families, 15 orthologs), which are involved in processing a variety of glycan substrates. One particular β-glucosidase in *B*. *breve* has been shown to degrade cellobiose and cellodextrin^38^. In addition, bifidobacterial β-glucosidases may be involved in targeting biologically active molecules such as natural phenols or flavonoids, being of particular relevance to the food and pharmaceutical industry^38^. For this reason and with the aim of finding possible substrates to a relatively high and uncharacterized β-glucosidases in our dataset (15 families), available plant-derived β-glucans and natural phenols (e.g. cellulose, pectin, salicin, esculin, arbutin, raftelin and amygdalin) were incorporated as substrates to be tested in our study (see below).

The third most abundant group of carbohydrate-active enzymes in *B*. *breve* is represented by β-galactosidases, which typically are members of the GH2 and GH42 families, allowing *B*. *breve* to grow on lactose, but also galactan and galacto-oligosaccharides (the former also requiring the action of an extracellular, GH53-family galactanase), and harvest galactose moieties from mucin- and milk-derived oligosaccharides^21,25,29,39,40^.

Taken together, the *B*. *breve* glycobiome clearly connects predicted saccharolytic activities with dietary substrates available in the infant gut. In fact, most of the carbohydrates present in the infant diet, up to weaning, are derived from milk and milk-derived products, which are substrates for bifidobacterial β-galactosidases. During the transition from a milk-based diet to solid foods (~6 months), fruits and cereals or vegetables are the first foods given to most infants^37^. Furthermore, starch, present in mashed potato, pasta or rice, may constitute an abundant substrate for bifidobacterial growth in the large intestine, if it escapes hydrolysis and consequent absorption in the small intestine. This is also consistent with our data showing the presence of a broad set of genes encoding GH13 members (Fig. 2A).

In order to explore substrate utilization by *B*. *breve* strains and linking such information with the presence of genes with a predicted function in carbohydrate metabolism, we determined growth profiles of the 20 *B*. *breve* strains on 77 different carbohydrate-containing compounds (Supplemental Table S3; Fig. 2B). The resulting growth profiles revealed that all strains grow well on seven carbohydrates (i.e. lactose, lactulose, maltose, melibiose, raffinose, glucose and GOS) (Fig. 2B). Of the remaining 70 tested carbohydrates, no growth was observed in 35 cases (45% of the total), which included various dietary/host-derived carbohydrates (e.g. xylose, XOS, glucuronic acid, galacturonic acid, glucans, arabinose, mannose, tagatose, fucose, 2-fucosyllactose and mucin), nucleosides (e.g. uridine, thymidine, adenosine, deoxycytidine), but also potential growth substrates for which no information had previously been collected for *B*. *breve* and bifidobacteria in general (e.g. saponin, cyclomaltoheptaose, γ-cyclodextrin, methyl-D-galactopyranoside, methyl-D-glucopyranoside, D-mandelonitrile-β-gentiobioside, methyl-D-glucopyranoside and myo-inositol) (Fig. 2B).

### GTM applied to carbon source utilization

**Figure 3:**
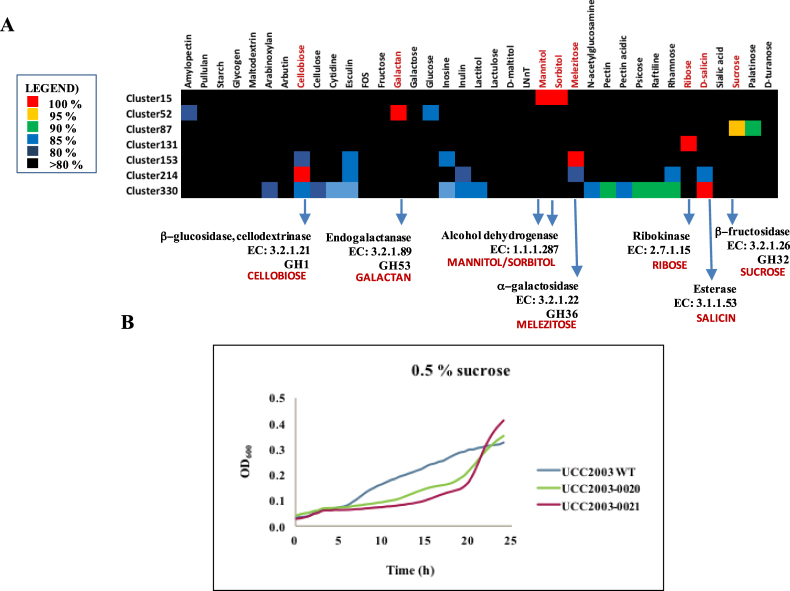
GTM analysis applied to carbohydrate utilization by *B*. *breve*. GTM in *B*. *breve* conducted for 337 presence/absence clusters and 37 potential growth substrates. Substrates were excluded from the displayed analysis if they supported growth of all tested strains or if no growth was observed for any of the strains (Supplemental Table S3). Panel A: Heatmap showing gene clusters that match growth patterns on seven substrates (cellobiose, galactan, mannitol/sorbitol, melezitose, ribose, salicin and sucrose). Panel B: Insertional mutagenesis and assessment of the sucrose utilization in *B*. *breve* UCC2003. Diagrams showing growth curves of *B*. *breve* UCC2003 and *B*. *breve* UCC2003-0020/21 insertional mutants in sucrose.

The combination of *in silico* predicted GH content and obtained *B*. *breve* growth profiles was used to establish associations between a range of substrates and possible gene(s) or cluster(s) responsible for their utilization, using a gene-trait matching (GTM) approach. GTM associations were established between 332 unique combinations or clusters of occurrence representing presence/absence of 785 gene families, and growth/no growth phenotype obtained for the 20 *B*. *breve* strains analysed. In this manner, we identified 23 gene families that exhibited a 100% match between their presence/absence and the ability/inability to grow on a particular carbohydrate (i.e. for cellobiose, galactan, mannitol, sorbitol, melezitose, ribose and salicin), in addition to a single gene family that exhibits a 95% correlation between its presence and the ability to grow on sucrose (Table 1, Fig. 3A).

**Table 1:** List of gene families (and surrounding regions with associated functions) returning positive hits in GTM analysis.

Cluster	Locus_tags	Annotation	GTM positive	PFAM	Reference
Cellobiose	Bbr_0104	Ketol-acid reductoisomerase/2-dehydropantoate 2-reductase		PF07991	Pokusaeva *et al*.^27^
	Bbr_0105	Cellodextrin transport system transcriptional regulator		PF13377	Pokusaeva *et al*.^27^
	Bbr_0106	Cellodextrin binding protein	X	PF13416	Pokusaeva *et al*.^27^
	Bbr_0107	Cellodextrin transport system permease protein CebF		PF00528	Pokusaeva *et al*.^27^
	Bbr_0108	Cellodextrin transport system permease protein CebG		PF00528	Pokusaeva *et al*.^27^
	Bbr_0109	Beta-glucosidase, cellodextrinase, glycosyl hydrolase	X	PF00232	Pokusaeva *et al*.^27^
	Bbr_0110	Ketol-acid reductoisomerase/2-dehydropantoate 2-reductase		PF07991	Pokusaeva *et al*.^27^
Galactan	Bbr_0417	Solute-binding protein of ABC transporter system for sugars		PF01547	O’Connell Motherway *et al*.^25^
	Bbr_0418	Permease protein of ABC transporter system for sugars		PF00528	O’Connell Motherway *et al*.^25^
	Bbr_0419	Permease protein of ABC transporter system for sugars		PF00528	O’Connell Motherway *et al*.^25^
	Bbr_0420	Beta-galactosidase		PF02449	O’Connell Motherway *et al*.^25^
	Bbr_0421	Transcriptional regulator, LacI family		PF13377	O’Connell Motherway *et al*.^25^
	Bbr_0422	Glycosyl hydrolases family 53, Endogalactanase	X	PF07745	O’Connell Motherway *et al*.^25^
Ribose	Bbr_1415	Ribokinase		PF00294	Pokusaeva *et al*.^26^
	Bbr_1416	Ribose transport system permease protein rbsD	X	PF05025	Pokusaeva *et al*.^26^
	Bbr_1417	D-ribose-binding protein rbsB		PF13407	Pokusaeva *et al*.^26^
	Bbr_1418	Ribose transport system permease protein rbsC		PF02653	Pokusaeva *et al*.^26^
	Bbr_1419	Ribose transport ATP-binding protein rbsA		PF00005	Pokusaeva *et al*.^26^
	Bbr_1420	Transcriptional regulator, LacI family		PF13377	Pokusaeva *et al*.^26^
	Bbr_1421	Conserved hypothetical membrane spanning protein	X	PF07690	Pokusaeva *et al*.^26^
	Bbr_1422	pfkB family carbohydrate kinase	X	PF00294	Pokusaeva *et al*.^26^
	Bbr_1432	Ribokinase	X	PF00294	Pokusaeva *et al*.^26^
Melezitose	Bbr_1855	Alpha-glucosidase		PF00128	O’Connell *et al*.^23^
	Bbr_1856	Alpha-galactosidase	X	PF05691	O’Connell *et al*.^23^
	Bbr_1857	Alpha-1,4-glucosidase		PF00128	O’Connell *et al*.^23^
	Bbr_1858	Permease protein of ABC transporter system for sugars		PF00528	O’Connell *et al*.^23^
	Bbr_1859	Permease protein of ABC transporter system for sugars		PF00528	O’Connell *et al*.^23^
	Bbr_1860	Solute binding protein of ABC transporter system for sugars	X	PF01547	O’Connell *et al*.^23^
Mannitol/Sorbitol	B7017_1839/NRBB01_1658	Alpha-acetolactate decarboxylase		PF03306	Bottacini *et al*.^22^
	B7017_1840 NRBB01_1659	Hypothetical membrane spanning protein		PF14256	Bottacini *et al*.^22^
	B7017_1841 NRBB01_1660	Aldehyde-alcohol dehydrogenase		PF00465	Bottacini *et al*.^22^
	B7017_1842/ NRBB01_1661	Transcriptional regulator, AraC family		PF12833	Bottacini *et al*.^22^
	B7017_1843/ NRBB01_1662	Alcohol dehydrogenase	X	PF08240	Bottacini *et al*.^22^
	B7017_1844/ NRBB01_1663	transporter, major facilitator family protein		PF07690	Bottacini *et al*.^22^
	B7017_1845/ NRBB01_1664	Transcriptional regulator, ROK family		PF00480	Bottacini *et al*.^22^
	B7017_1846/ NRBB01_1665	putative glyoxalase family protein		PF12681	Bottacini *et al*.^22^
	B7017_1847/ NRBB01_1666	ribitol transporter		PF07690	Bottacini *et al*.^22^
	B7017_1848/ NRBB01_1667	Alcohol dehydrogenase	X	PF08240	Bottacini *et al*.^22^
Salicin	NRBB52_0572	Alcohol dehydrogenase		PF08240	Uncharacterized
	NRBB52_0573	Hypothetical protein		PF13173	Uncharacterized
	NRBB52_0574	Hypothetical protein		PF02518	Uncharacterized
	NRBB52_0575	Carbohydrate esterase	X	PF03629	Uncharacterized
	NRBB52_0576	Hypothetical protein	X	No hit	Uncharacterized
	NRBB52_0577	Beta-glucosidase	X	PF00232	Uncharacterized
	NRBB52_0578	Oligopeptide-binding protein (oppA)		PF00496	Uncharacterized
	NRBB52_0579	Oligopeptide transport system permease protein oppB		PF00528	Uncharacterized
	NRBB52_0580	Permease protein of ABC transporter system for peptides		PF00528	Uncharacterized
	NRBB52_0581	Oligopeptide transport ATP-binding protein oppD		PF00005	Uncharacterized
	NRBB52_0582	Oligopeptide transport ATP-binding protein oppF		PF00005	Uncharacterized
	NRBB52_0583	Beta-galactosidase		PF00005	Uncharacterized
	NRBB52_0584	Transcriptional regulator		PF13377	Uncharacterized
Sucrose	Bbr_0018	Hypothetical membrane spanning protein		PF04854	Uncharacterized
	Bbr_0019	Transcriptional regulator, LacI family		PF13377	Uncharacterized
	Bbr_0020	Beta-fructosidase or sucrose-6-phosphate hydrolase	X	PF00251	Uncharacterized
	Bbr_0021	Solute-binding protein ABC transporter	X	PF13416	Uncharacterized
Tetracycline	BB139W423_0392	Transposase		PF00665	Uncharacterized
	BB139W423_0393	Ribosomal protection tetracycline resistance protein	X	PF00009	Uncharacterized
Erythromycin	NRBB51_1106	Transposase		PF10551	Uncharacterized
	NRBB51_1107	Transposase		PF00872	Uncharacterized
	NRBB51_1108	Dimethyladenosine transferase (ErmX)	X	PF00398	Uncharacterized
	NRBB51_1109	Transposase		PF10551	Uncharacterized
	NRBB51_1110	Transposase		PF00872	Uncharacterized
	NRBB51_1111	Dimethyladenosine transferase (ErmX)	X	PF00398	Uncharacterized
	NRBB51_1112	Transposase		PF10551	Uncharacterized
	NRBB51_1113	Transposase		No hit	Uncharacterized
	NRBB51_1114	Dimethyladenosine transferase (ErmX)	X	PF00398	Uncharacterized
	NRBB51_1115	Transposase		PF10551	Uncharacterized
	NRBB51_1116	Transposase		PF01610	Uncharacterized
Aminoglycosides	CNCMI4321_0985	Aminoglycoside phosphotransferase (APH)	X	PF01636	Uncharacterized
	CNCMI4321_0986	N-acetyltransferase (AAC)	X	PF00583	Uncharacterized
	CNCMI4321_0987	Aminoglycoside adenylyltransferase (ANT)	X	PF04439	Uncharacterized
EPS biosynthesis	Bbr_0430	Undecaprenyl-phosphate galactosephosphotransferase	X	PF02397	Fanning *et al*.^55^
	Bbr_0431	Protein tyrosine phosphatase	X	PF01451	Fanning *et al*.^55^
	Bbr_0432	Transposase		PF01695	Fanning *et al*.^55^
	Bbr_0433	Transposase		PF00665	Fanning *et al*.^55^
	Bbr_0434	Oligosaccharide repeat unit transporter (flippase)	X	PF01943	Fanning *et al*.^55^
	Bbr_0444	Membrane spanning EPS biosynthesis protein (flippase)	X	PF01943	Fanning *et al*.^55^
	Bbr_0435	Beta-1,6-N-acetylglucosaminyltransferase		PF02485	Fanning *et al*.^55^
	NRBB56_0458	UDP-galactopyranose mustase		PF03275	Uncharacterized
	NRBB50_0526	Hypothetical thiamine pyrophosphate enzyme TPP	X	PF02776	Uncharacterized
	NRBB50_0514	Hypothetical membrane spanning protein (polymerase?)	X	No hit	Uncharacterized
	Bbr_0436	Hypothetical membrane spanning protein (polymerase)	X	PF14897	Fanning *et al*.^55^
	Bbr_0450	Membrane spanning protein (polymerase)	X	PF14897	Fanning *et al*.^55^
	Bbr_0437	Acetyltransferase		PF00132	Fanning *et al*.^55^
	Bbr_0446	Acetyltransferase (cell wall biosynthesis)		PF00132	Fanning *et al*.^55^
	Bbr_0451	Acyltransferase		PF01757	Fanning *et al*.^55^
	Bbr_0438	Glycosyltransferase	X	PF00535	Fanning *et al*.^55^
	Bbr_0445	Glycosyltransferase	X	PF00535	Fanning *et al*.^55^
	Bbr_0448	Glycosyltransferase	X	PF00535	Fanning *et al*.^55^
	Bbr_0443	Glycosyltransferase	X	PF00534	Fanning *et al*.^55^
	Bbr_0441	Capsular polysaccharide biosynthesis protein	X	PF00534	Fanning *et al*.^55^
	Bbr_0442	Capsular polysaccharide biosynthesis protein	X	PF00534	Fanning *et al*.^55^
	Bbr_0439	Capsular polysaccharide biosynthesis protein	X	PF05704	Fanning *et al*.^55^
	Bbr_0440	Polysaccharide biosynthesis protein	X	PF14393	Fanning *et al*.^55^
	Bbr_0447	Conserved hypothetical protein		PF04230	Fanning *et al*.^55^
	Bbr_0449	Hypothetical membrane spanning protein		No hit	Fanning *et al*.^55^
	Bbr_0452	Hypothetical protein		No hit	Fanning *et al*.^55^
	Bbr_0453	Transposase		PF01695	Fanning *et al*.^55^
	Bbr_0454	Conserved hypothetical protein		No hit	Fanning *et al*.^55^
	Bbr_04^55^	Transposase		PF01695	Fanning *et al*.^55^
	Bbr_0456	Transposase		PF00665	Fanning *et al*.^55^
	Bbr_0457	Transposase		PF00665	Fanning *et al*.^55^
	Bbr_0458	Hypothetical protein		No hit	Fanning *et al*.^55^
	Bbr_0459	Conserved hypothetical protein		PF07693	Fanning *et al*.^55^
	Bbr_0460	Hypothetical membrane spanning protein		No hit	Fanning *et al*.^55^
	Bbr_0461	Hypothetical protein		No hit	Fanning *et al*.^55^
	Bbr_0462	Transposase		PF01695	Fanning *et al*.^55^
	Bbr_0463	Transposase		PF00665	Fanning *et al*.^55^
	Bbr_0464	Hypothetical protein		PF14280	Fanning *et al*.^55^
	Bbr_0465	Hypothetical protein		PF14253	Fanning *et al*.^55^
	Bbr_0466	Hypothetical protein		No hit	Fanning *et al*.^55^
	Bbr_0467	Conserved hypothetical protein with a helix-turn-helix motif		PF01381	Fanning *et al*.^55^
	Bbr_0468	Hypothetical protein		No hit	Fanning *et al*.^55^
	Bbr_0471	Hypothetical protein		PF12686	Fanning *et al*.^55^
	Bbr_0472	Conserved hypothetical membrane spanning protein		PF13425	Fanning *et al*.^55^
	Bbr_0473	Conserved hypothetical protein		No hit	Fanning *et al*.^55^
	Bbr_0474	Chain length regulator		PF13614	Fanning *et al*.^55^

These GTM results therefore confirm and extend a previous analysis on a much smaller number of strains and carbohydrates^22^, while it furthermore validates the GTM approach in identifying the genetic requirements for growth on certain carbohydrates^22,23,25–27^. More specifically, GTM identified associations between carbohydrate-mediated growth and gene clusters known to be involved in the utilization of cellobiose, galactan, mannitol, sorbitol, melezitose, ribose, salicin and sucrose (Supplemental Fig. S1).

It is worth noting that involvement of particular genetic clusters in the utilization of certain substrates had previously been demonstrated in *B*. *breve* UCC2003 using classical techniques (Table 1). An exception to this is the sorbitol/mannitol utilization cluster, which was identified by GTM employing a smaller number of *B*. *breve* strains^22^. Overall, the GTM method adopted here not only allowed us to corroborate our previous findings, but also facilitated the identification of genes involved in sucrose and salicin metabolism.

*B*. *breve* NRBB52 is the only strain (of the 20 analysed) that is able to grow on salicin as its sole carbon source. This ability is associated with the presence of a gene cluster, which is unique to this strain, and which contains genes predicted to encode an esterase (NRBB52_0575), a β-glucosidase (NRBB52_0577), an ABC-type transport system (NRBB52_0578-82) and a β-galactosidase (NRBB52_0583) (Supplemental Fig. S1). Esterases and β-glucosidases are known to be involved in the enzymatic degradation of salycilates^41^, and this NRBB52-associated gene cluster was indeed shown to be required for utilization of salicin.

In the case of sucrose, 13 out of the 20 analysed *B*. *breve* strains were shown to utilize this disaccharide as their sole carbon source. GTM association revealed (for 12 out of these 13 strains) two previously uncharacterized genes encoding a predicted β-fructosidase or sucrose-6-phosphate hydrolase (Bbr_0020) and a solute binding protein of an ABC transporter system (Bbr_0021). It has previously been shown that bifidobacterial β-fructofuranosidases constitute enzymes involved in the breakdown of sucrose and sugars with fructose-containing moieties^28^. In our case we also noticed that the absence of this particular cluster in four cases corresponds to lack of growth on sucrose as well as palatinose and, though to a lesser extent, growth on turanose and fructose, thus supporting the notion that this cluster is involved in the utilization of a diverse range of fructose-containing substrates (Fig. 2B).

In order to verify the involvement of the genes encoding the predicted β-fructosidase (designated here as *bfrA*, corresponding to locus tag Bbr_0020) and solute binding protein (designated here as *brfB*, corresponding to locus tag Bbr_0021) in the utilization of fructose-containing moieties, they were targeted for insertional mutagenesis in *B*. *breve* UCC2003. Wild type *B*. *breve* UCC2003 and the isogenic strains carrying a mutation in either *bfrA* or *bfrB* (designated here as *B*. *breve* UCC2003-0020 and *B*. *breve* UCC2003-0021, respectively) were tested for their ability to grow on sucrose. As can be observed from the obtained growth profiles (Fig. 3B) both insertion mutant strains exhibited reduced growth on sucrose, thus confirming the GTM predictions. Notably, growth on sucrose was not fully eliminated, probably because sucrose may be metabolized by alternative routes in *B*. *breve* UCC2003 (e.g. a sucrose phosphorylase Bbr_100, an ABC transporter Bbr_0026-27, an additional β-fructofuranosidase Bbr_1324 and a fructose phosphotransferase PTS system Bbr_1594)^23,28,42^. Of the 35 substrates for which differential growth was observed among *B*. *breve* strains, some of these (e.g. starch and starch-like polysaccharides, lacto-*N*-neotetraose, and galactose), did not return any useful positive matches when employing GTM. Apparently, utilization of certain carbohydrates may be influenced by factors other than the simple presence/absence of genes (e.g. gene regulation, but also involvement of multiple distinct pathways or point mutations), and in such cases a different approach will be required to identify the genes involved.

### GTM applied to antibiotic resistance

**Figure 4:**
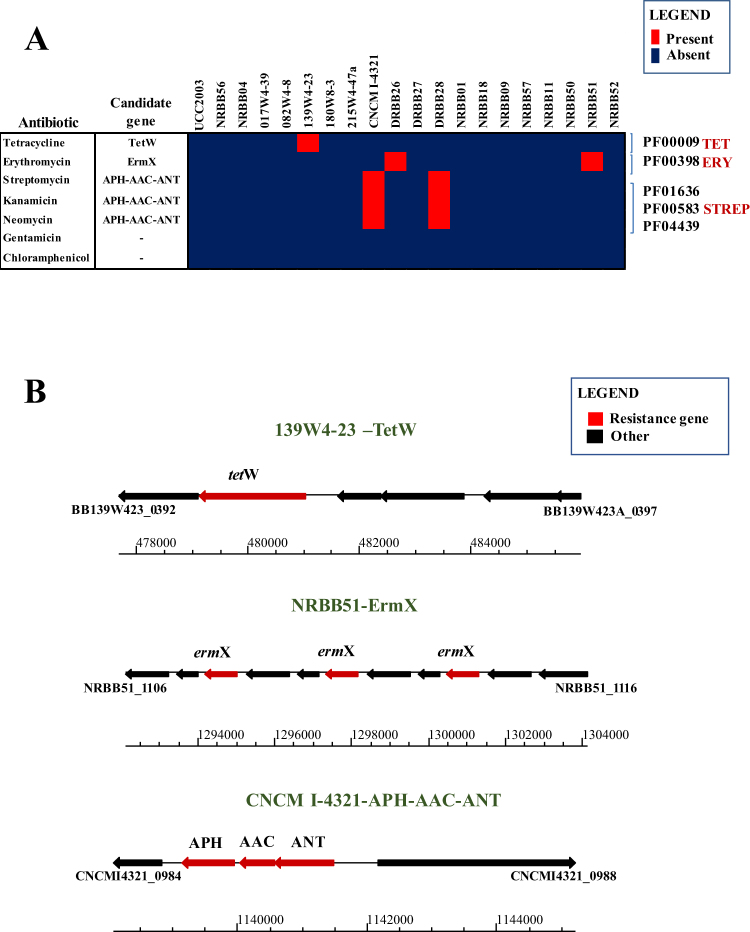
GTM analysis applied to antibiotic resistance in *B*. *breve*. Antibiotic resistance markers in the 20 *B*. *breve* genomes with corresponding phenotypic assays. Panel A: Heatmap representing presence /absence of identified antibiotic resistance markers across 20 *B*. *breve* strains matching the observed phenotype. Panel B: Locus map representing the surrounding regions of the identified antibiotic resistance markers (e.g. tetracycline, erythromycin and aminoglycoside resistance) returning positive match in GTM.

The use of GTM to discover carbohydrate utilization gene clusters in *B*. *breve* encouraged us to apply this method to antibiotic resistance phenotypes. In order to generate our phenotypic dataset, the 20 *B*. *breve* strains were tested for their sensitivity to a range of antibiotics (Supplemental Table S4). Based on analysis tetracycline (Tet), erythromycin (Ery) and aminoglycoside (streptomycin; Str) resistance phenotypes were detected for *B*. *breve* 139W4-23, *B*. *breve* NRBB51/DRBB26 and *B*. *breve* CNCM I-4321/DRBB28, respectively (Fig. 4A).

In order to assess whether the presence/absence of antibiotic resistance genes is linked to a corresponding resistance phenotype, GTM analysis was employed using the same approach as described for carbohydrate utilization. In the case of Tet resistance, a clear *tet*W homolog, conferring ribosomal protection from translation inhibition by Tet^43^, was found in the genome of *B*. *breve* 139W4-23 (locus tag BB139W423_0393), thus representing a perfect match with the observed phenotype (Fig. 4B). In *B*. *breve* 139W4-23 the *tetW* homolog is surrounded by multiple transposases, suggesting acquisition by horizontal transfer.

For erythromycin resistance, multiple copies of an *erm*X gene were found in the genome of *B*. *breve* NRBB51 (three copies) and *B*. *breve* DRBB26 (two copies), and were shown to be interleaved by predicted transposase-encoding genes (Fig. 4B). Erythromycin resistance in bifidobacteria has previously been described in *B*. *thermophilum*, where the *erm*X gene is flanked by Tn5432-like transposons^44^, similarly with what we observed in this strain. Employment of long-reads from Pacbio SMRT sequencing (average read length 14 Kb) accurately identified multiple repetitions of the Tn5432-*erm*X system within a 10 Kb chromosomal region, otherwise not easily detectable with the sole employment of short-read sequencing which generally results in a shorter and gapped consensus. The erythromycin resistance observed for *B*. *breve* NRBB51 represents a positive GTM match with the presence of the Tn5432-*erm*X system in this strain. Closer inspection of the *erm*X locus in *B*. *breve* DRBB26 revealed the presence of a structural variation in this genome resulting from the reshuffling of the two transposases preceding the *erm*X genes, which may have significantly lowered the erythromycin resistance of this strain (<0.5 μg/ml). Interestingly, the presumed acquisition of this locus through horizontal gene transfer (HGT) is also corroborated by the finding that the *erm*X locus in *B*. *breve* NRBB51 is located on a putative integrative conjugative element (data not shown). To confirm that *erm*X of NRBB51 (corresponding to locus tag NRBB51_1114) confers erythromycin resistance this gene was cloned into pNZ44 resulting in plasmid pNZ44-Ery, of which introduction into the erythromycin-sensitive *B*. *breve* UCC2003 resulted in the expected antibiotic resistance phenotype (Table 2).

**Table 2:** Assessment of antibiotic resistance markers.

	Antibiotic	Highest level tested (μg/ml)	UCC2003 pNZ44	UCC2003 pNZ44-Str (0985-87)	UCC2003 pNZ44-0985	UCC2003 pNZ44-0986	UCC2003 pNZ44-0987	UCC2003 pNZ44-Ery (1114)
Aminoglycosides	Amikacin	256	Res	Res	Res	Res	Res	NA
	Kanamycin	256	Res	Res	Res	Res	Res	NA
	Tobramycin	128	Res	Res	Res	Res	Res	NA
	**Streptomycin**	1024	Sen	Res*	Sen	Sen	Res*	NA
	Gentamicin	24	Sen	Sen	Sen	Sen	Sen	NA
	Netilmicin	48	Res	Res	Res	Res	Res	NA
Erythromycin	**Erythromycin**	256	Sen	NA	NA	NA	NA	Res*

^*^Confirmation of GTM Predictions for streptomycin and erythromycin. Res = resistant strain to the level tested; Sen = sensitive strain to the level tested; NA = not applicable.

For aminoglycoside resistance, GTM analysis pointed to a genetic locus containing three predicted aminoglycoside transferases in *B*. *breve* CNCM I-4321 (corresponding to locus tags CNCMI4321_0985-87) (Fig. 4B) and *B*. *breve* DRBB28 (locus tags DRBB28_1389-91). In both cases, these genes are present within a chromosomal region with a lower G + C content (40%) as compared to the average G + C content (59%), suggesting HGT acquisition. Based on analyses employing the comprehensive antibiotic resistance CARD/ARDB (https://card.mcmaster.ca) and PFAM (http://pfam.xfam.org) databases, this locus appears to encode three enzymes each responsible for a particular modification (Table 1, Fig. 4A) that on its own or in combination may inactivate one or more aminoglycosides.

Based on the predicted functions, cloning of the genes within the CNCMI4321_0985-87 locus either together or individually was performed and their possible activity against a range of aminoglycosides was assessed. The result of the cloning experiments specifically assigned streptomycin resistance to the predicted nucleotidyl-transferase encoding gene (CNCMI4321_0987) (Table 2), thus resulting in the identification of several antibiotic selection markers, which in turn may be used to develop novel genetic tools specific for bifidobacteria.

### GTM applied to EPS production

In order to extend our GTM analysis to a third phenotype, we investigated whether the presence/absence of certain exopolysaccharide (EPS)-biosynthesis related genes would result in a corresponding EPS production phenotype and furthermore deduce a possible consensus for *B*. *breve* EPS “producers” and “non-producers”.

Gene content comparison of the EPS region, when applied to 20 fully sequenced representatives of *B*. *breve*, revealed that this locus, despite being always present at the same chromosomal position across the species, is one of the most variable genomic regions, with a very substantial difference in gene content and size across strains ranging between 5 and 58 Kb (Supplemental Fig. S2).

**Figure 5:**
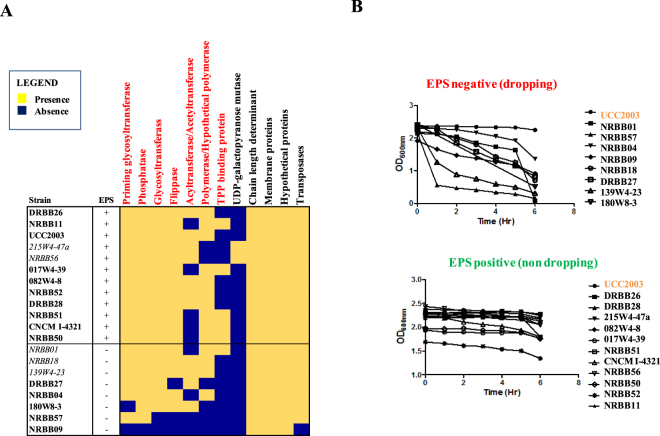
GTM analysis applied to EPS production by *B*. *breve*. GTM applied to EPS production phenotype in *B*. *breve*. Panel A: Heatmap representing the EPS-associated genetic functions of which presence matched the observed EPS production phenotype (red) across the assessed *B*. *breve* strains. Five strains showing discrepant genotype/phenotype association are italicized. Panel B: EPS production phenotypic assay. Non-EPS-producers clearly show apparent reduction in OD as they ‘sediment’ to the bottom of the tube, while EPS producers do not exhibit such a sedimentation phenotype.

As previously described for certain bifidobacterial strains (among which are *B*. *breve* UCC2003, *B*. *longum* subsp. *longum* 35624^TM^ and *B*. *animalis* subsp. *lactis* A1dOxR), bifidobacterial derivatives that do not produce EPS can be discriminated from their EPS-producing parental strains because the former strains sediment when cultivated in liquid growth media^45–48^. This phenotypic assay was carried out on the 20 *B*. *breve* strains and indicated that in 60% of the cases (12 representatives) EPS production did occur, while for the remaining 40% of the strains (8 representatives) no EPS appeared to be produced as such strains exhibit a clear sedimentation phenotype in liquid medium (Fig. 5B).

By a first comparison of the phenotypic assay with the size of the EPS region across *B*. *breve*, no significant correlation between EPS production and the size of the locus was observed. However, our data delineated that *B*. *breve* EPS loci longer than 50 Kb are more likely to be complete and contain all necessary functions required for EPS biosynthesis, while loci < 30 Kb probably constitute incomplete and non-functional systems (Supplemental Fig. S2). In the case of EPS regions of a size between 30 and 50 Kb, either EPS “producers” or “non-producers” were observed, suggesting that EPS biosynthesis gene clusters of this size may still contain all genetic information for EPS production. Furthermore, a complete deletion of this region (*B*. *breve* NRBB09) indeed corresponded to the expected EPS-negative phenotype.

In order to further investigate which combination of presence/absence of genes would result in a successful capsule biosynthesis, a comparative analysis of the genes present in this region was performed across strains and compared with the observed phenotype in a GTM approach. The analysis elucidated that a given *B*. *breve* strain can be classified as an EPS “producer” if it possesses the following characteristics: *a)* locus size > 30 Kb, *b)* at least three glycosyltransferase(GT)-encoding genes, *c)* an additional GT-encoding gene typically found at the beginning of the locus and representing the priming glycosyltransferase, *d)* a gene encoding a flippase, *e)* a gene encoding a tyrosine kinase or chain length determinant, *f)* an acetyltransferase often found in the vicinity of a polymerase-encoding gene or, alternatively, a thiamine pyrophosphate (TPP) binding protein always present in association with a membrane spanning protein which presumably represents the polymerase (Figs 5A,C). In summary, the GTM analysis allowed to explain the EPS production of 10 out of 12 strains (Fig. 5A and C). Notably, strains 215W4-47a and NRBB56 phenotypically behave as EPS “producers”, though our genetic analysis did not identify a candidate gene for a polymerase in the associated EPS locus. Other genetic functions, such as those encoding putative acetyltransferase, UDP-galactopyranose mutase and chain length-determining activities, did not appear to be able to discriminate “producers” from “non-producers” as they either occur in a limited number of strains or all strains (Fig. 5A). Therefore, our comparative and GTM analysis when applied to EPS production in *B*. *breve* defined gene functions which can be used for *in silico* discrimination between potential EPS “producers” and “non-producers”, thus facilitating the *in silico* screening of *B*. *breve* genomes for this phenotypic trait.

## Conclusions

Comparative genome analysis represents a powerful bioinformatics tool to assess gene distribution across members of a given species. If combined with pan-genomic extrapolations, gene comparisons may also allow identification of genes that are responsible for strain diversification. The presence of gene families that are variably present across members of a given species can be used as a starting point for phenotypic investigations using GTM. Our efforts to assess 20 *B*. *breve* representatives for a number of different phenotypes generated novel information about the implications of the genetic diversity observed for this species (e.g. carbohydrate utilization capabilities and EPS biosynthesis).

The main advantage of GTM as compared to classical approaches, generally based on single strain investigation and not employing comparative genomic analysis, is that the first method is a much faster, yet accurate method to pin-point variably distributed genes, even within large genomic regions, being responsible for different phenotypic traits. Our findings indeed show that GTM can be used to identify genes involved in carbohydrate utilization in *B*. *breve* and by extension (bifido)bacteria in general.

Expansion of this analysis to a different phenotypes allowed the identification of novel antibiotic resistance markers which may be useful to develop novel cloning vectors specific for *B*. *breve* or bifidobacteria in general. Moreover, the application of GTM to a more complex phenotype such as capsule biosynthesis constituted a challenge for the analysis, in particular because EPS production is the result of a combination of presence and co-occurrence of multiple genes. Furthermore, in this case the comparative genome analysis and GTM allowed us to identify genetic functions linked to the observed phenotype, thereby facilitating the distinction between EPS “producers” and “non-producers”.

Taken together, our study shows that comparative genome analysis can be integrated with phenotypic investigations in order to identify candidate genes responsible for various phenotypic traits, thereby generating new information on the unique genetic features of members of this bifidobacterial species.

## Materials and Methods

### *In silico* comparative analyses and pan-genome computation

We selected the prototype strain *B*. *breve* UCC2003^31^ as a reference for our study, supplemented by 19 *B*. *breve* representatives (Supplemental Table S1) from a collection of *B*. *breve* human isolates, that had been sequenced in a previous study^30^. Comparative genome analyses and alignments for these 20 selected *B*. *breve* strains were carried out at protein level using “all-against-all”, bi-directional BLASTP alignments^49^ (cut-off: E-value < 0.0001, with at least 50% identity across at least 50% of either protein sequence). Identified ORFs were organized, based on these BLASTP outputs, in functionally related protein families employing the Markov Cluster Algorithm (MCL) implemented in the mclblastline pipeline v12-0678^50^. Comparative genome analyses then allowed the classification of the obtained gene families into either the core- or the dispensable-genome based on the presence of homologs in either all strains or in a subset of them, respectively. Pan-genome computation was performed using the online available PGAP v1.2 pipeline, which uses the Heap’s law pan-genome model; genes were clustered using the GF (Gene Family) method implemented in the pipeline^51^.

### Genotype/phenotype associations among *B*. *breve* strains

Carbohydrate-dependent growth profile analysis of *B*. *breve* strains was performed employing potential saccharide and saccharide-containing growth substrates (77 different growth substrates; Supplemental Table S3). For growth analysis, a 5% (w/v) stock solution of a given substrate (Supplemental Table S3) was prepared using distilled water. The obtained solution was filter sterilized using a 0.45 μm membrane filter and stored at 4 °C until required. To test the growth potential of each *B*. *breve* strain, modified de Man Rogosa and Sharpe (mMRS) medium was used, formulated based on the absence of any carbohydrate source (other than those to be tested). The medium was freshly prepared from first principles^52^ and supplemented with 0.5% (v/v) of an individual growth substrate solution and 0.05% (v/v) of L-cysteine HCl as essential nitrogen source for bifidobacterial growth. To this medium, 1% (v/v) of an overnight *B*. *breve* culture previously cultivated in 1% glucose or lactose, depending on the strain, was added. In some cases, in particular for poorly soluble carbohydrates (i.e. arabinoxylan, galactan, glycogen, starch, amylopectin, pullulan and maltodextrin), the substrate was directly added to mMRS at a final concentration of 0.5% prior to autoclaving at 121 °C for 15 minutes. Medium without any supplemented carbohydrate was used as a negative control for each experiment, while medium with lactose was used as a positive control. Cultures were grown anaerobically at 37 °C and optical density measurements were recorded at OD_600nm_ at regular intervals within 24 hours. Growth profiles were obtained using an automated microplate spectrophotometer (MultiScan FC Reader, Thermo Fisher). In the case of poorly soluble carbohydrates OD_600nm_ measurements were taken manually using a UV-1280 spectrophotometer (Shimadzu Corporation, Kyoto, Japan). Results were presented as a binary heatmap where growth/no-growth patterns were deduced based on the following optical density cut-off values at 12 hours of incubation: (no growth = OD_600_ < 0.2; good growth = OD_600_ > 0.3; intermediate growth = OD_600_ > 0.2 and < 0.3), slightly adjusted from a previous publication^39^.

An *in silico* genotype/phenotype or GTM analysis was performed to associate presence/absence of specific gene families from the variome with a phenotype. Based on the fact that carbohydrate utilization capabilities are conferred by the presence of specific gene clusters, we organized presence/absence of gene families (retrieved by comparative genome analysis) and growth/no-growth phenotype, and the selection of candidates was performed for each phenotype on an individual basis, as described previously^22,53^. In order to apply GTM analysis to carbon source utilization, identified gene families were filtered to exclude those that are present in all strains, as not contributing to strain diversification. Elements of the *B*. *breve* variome related to bacteriophage, certain mobile elements (e.g. integrated episomes and prophages) and phage-defence mechanisms (e.g. CRISPR/Cas and restriction-modification systems), which are not assumed to be associated with carbon source utilization, were also removed from the GTM analysis.

The gene families obtained in this manner were further clustered into unique combinations of occurrence across strains and a binary matrix was then deduced from the dataset (values 0 for absence and 1 for presence of a cluster). The thus generated “genotype” matrix contains 337 clusters as rows and 20 strains as columns. The same approach was adopted to cluster the obtained fermentation profiles resulting in a binary matrix constituting the “phenotype”. For this purpose a lower limit OD_600nm_ of 0.3 was used as cut-off value to discriminate between substrates that did or did not support growth of a given strain (values 1 and 0, respectively). The resulting “phenotype” binary matrix contained 37 carbohydrates (for which differential growth was observed) as rows and 20 strains as columns, the latter organized in the same order as in the genotype matrix.

Percentage association between the presence of a particular cluster and a growth phenotype was represented in a heatmap. The obtained positive matches (>95% of match between “genotype” and “phenotype”) and adjacent genomic regions were further inspected by BLAST analysis, and compared with information retrieved from the Cazy database (http://www.cazy.org), EC (Enzyme Classification) database (http://www.expasy.ch/enzyme) and PFAM (http://pfam.sanger.ac.uk) alignments.

### Insertional mutagenesis in *B*. *breve* UCC2003

Experimental validation of the GTM prediction in relation to sucrose utilization was performed through insertional mutagenesis in *B*. *breve* UCC2003. Internal fragments of the predicted β-fructosidase (Bbr_0020) and the solute binding protein (Bbr_0021) genes (410 bp and 374 bp, respectively) were amplified by PCR using *B*. *breve* UCC2003 chromosomal DNA as template and specific primers (Supplemental Table S5). The insertional mutants *B*. *breve* UCC2003-0020 and *B*. *breve* UCC2003-0021 were generated according to a previously described method^54^. Site specific recombination and integration of the *tet*W gene in the correct chromosomal locations were confirmed by colony PCR using appropriate primers (Supplemental Table S5).

### Genotype/phenotype association applied to antibiotic resistance

Antibiotic resistance or susceptibility was determined by spread plating 100 µl of a particular *B*. *breve* strain on reinforced clostridial agar (RCA), on which antibiotic disks or strips (Supplemental Table S4) had been placed, followed by anaerobic incubation at 37 °C for 24 h and then scoring growth/no growth around disks. To identify antibiotic resistance genes in *B*. *breve*, we applied GTM to compare presence/absence of gene families with antibiotic susceptibility results. Positive GTM hits were further assessed using the Comprehensive Antibiotic Resistance Database (CARD/ARDB) (https://card.mcmaster.ca). Resistance gene searches were supported by BLASTP^49^ alignments using more stringent criteria than those used for the comparative analysis, in order to reduce the occurrence of false positives (cut-off: E-value < 0.0001, with at least 80% of identity across at least 70% of either protein sequence).

### Plasmid construction and cloning of antibiotic resistance genes

To validate the predicted functionality of genes in erythromycin resistance, chromosomal DNA from *B*. *breve* NRBB51 was used as a template for PCR amplification of the *erm*X gene (corresponding to locus tag NRBB51_1114) using Q5 DNA polymerase and primer pair 1114 F and 1114 R (Supplemental Table S5), in which PstI and HindIII sites had been incorporated so as to facilitate ligation to the similarly digested pNZ44. The ligation was introduced into electrocompetent *L*. *lactis* NZ9000 by electroporation and transformants were selected on GM17 agar plus 5 μg/ml chloramphenicol, resulting in pNZ44-Ery. Plasmid pNZ44-Ery was then introduced into electrocompetent *B*. *breve* UCC2003 by electroporation and the resulting recombinant strain named *B*. *breve* UCC2003-pNZ44-Ery was checked for erythromycin resistance (Table 2).

To validate the predicted involvement of genes in conferring streptomycin (Str) resistance, chromosomal DNA from *B*. *breve* CNCM I-4321 was used as template for PCR amplification of the suspected Str-resistance cassette (encompassing three genes, corresponding to locus tags CNCMI4321_0985-87) using Q5 DNA polymerase, and primer pair 0985-87 F and 0985-87 R (Supplemental Table S5), which included PstI and HindIII sites to facilitate ligation to similarly digested pNZ44. The individual three genes of the Str-resistance cassette were also assessed using specific primer pairs 0985 F and 0985 R, 0986 F and 0986 R, and 0987 F and 0987 R (Supplemental Table S5). Ligations were introduced into electrocompetent *L*. *lactis* NZ9000 by electroporation and transformants were selected on GM17 agar supplemented with 5 μg/ml chloramphenicol and checked for the correct insert by sequencing. This resulted in plasmid pNZ44-Str (carrying the entire resistance locus CNCMI4321_0985-87) and pNZ44-0985, pNZ44-0986, pNZ44-0987 (carrying individual genes, plasmid names correspond to locus tag numbers). The resulting four plasmids were then introduced into *B*. *breve* UCC2003 by electroporation, and the resulting recombinant strains, named *B*. *breve* UCC2003-pNZ44-Str, *B*. *breve* UCC2003-pNZ44-0985, *B*. *breve* UCC2003-pNZ44-0986, and *B*. *breve* UCC2003-pNZ44-0987 were then checked for resistance to a range of aminoglycosides, including streptomycin (Table 2). *B*. *breve* UCC2003-pNZ44 was used as a negative control for these experiments.

### Genotype/phenotype association as applied to EPS production

Exopolysaccharide (EPS) production was determined by first culturing 100 µl of a particular *B*. *breve* culture in mMRS media anaerobically at 37 °C for ~ 16 h. These cultures were then thoroughly resuspended to bring cells in full suspension. Incubation was then continued without agitation and cell sedimentation phenotype was assessed by hourly OD_600nm_ measurements for each of the *B*. *breve* strains over a 6-h time period. An observed drop in optical density (OD_600nm_ value substantially decreasing within the first 3 hours) was associated with a sedimentation phenotype, which in turn was presumed to correspond to an EPS negative phenotype. In order to establish associations between suspected EPS production phenotype and the presence of an intact gene cluster responsible for EPS biosynthesis, all genes contained in the EPS cluster of *B*. *breve*^22,55^ were first extracted and their presence/absence from comparative analysis was compared with the obtained phenotypic data in a gene-trait matrix-based approach analogous to the one described above.

### Data deposition

All the sequences used for our analysis have been retrieved from GenBank database with the following accession numbers: CP000303.1, CP021384, CP021389, CP021386, CP021387, CP021388, CP023193, CP021391, CP021392, CP021393, CP021394, CP021559, CP021390, CP021552, CP021553, CP021554, CP021555, CP021557, CP021556, CP021558.

## Electronic supplementary material

Supplementary material
