# Supplementary material for: Comparative genomics and genotype-phenotype associations in Bifidobacterium breve
Source: Sci Rep. 2018 Jul 13;8:10633. doi: 10.1038/s41598-018-28919-4 (PMC6045613; doi:10.1038/s41598-018-28919-4)

Comparative genomics and genotype-phenotype associations in *Bifidobacterium breve*.

Francesca Bottacini[1], Ruth Morrissey[1], Maria Esteban Torres[1], Kieran James[1,2], Justin van Breen[1], Evgenia Dikareva[1], Muireann Egan[1], Jolanda Lambert[3], Kees van Limpt[3], Jan Knol[3,4], Mary O'Connell Motherway[1] and Douwe van Sinderen[1,2]*.

[1] APC Microbiome Ireland and [2] School of Microbiology, University College Cork, Western Road, Cork, Ireland

[3] Danone Nutricia Research, Utrecht, The Netherlands

[4] Laboratory of Microbiology, Wageningen University, Wageningen, The Netherlands

* Corresponding author.

Corresponding author email address: d.vansinderen@ucc.ie

Supplementary Material.

Figure S1. Gene clusters returning positive hit in GTM analysis.

Locus maps of the seven GTM positive hits with surrounding regions on the chromosome. From these maps one can discern how growth on substrates corresponds to the insertion of one or multiple genes within the *B. breve* chromosome.

Figure S2. EPS production in *B. breve*.

Locus map representing the EPS region in *B. breve* across the 20 *B. breve* representatives ordered by decreasing locus size. In red and green are indicated those representatives showing an "EPS negative" and "EPS positive" phenotype in experimental assay.

Table S1. List of *Bifidobacterium breve* strains used for GTM analysis.

	Organism/Name	Origin (Reference)	Accession number	Size (Mb)	ORF number
1	*Bifidobacterium breve* UCC2003	Infant isolate (Breast Fed) [39]	CP000303	2422680	1854
2	*Bifidobacterium breve* NRBB01	Infant isolate [40]	CP021384	2269404	1902
3	*Bifidobacterium breve* NRBB57	BbC50, infant [40]	CP021389	2510381	2125
4	*Bifidobacterium breve* NRBB04	Infant isolate, ATCC 15698 [40]	CP021386	2324647	1874
5	*Bifidobacterium breve* NRBB09	Infant isolate (Breast Fed) Subject 3 [40]	CP021387	2265557	1881
6	*Bifidobacterium breve* NRBB11	Infant isolate (Breast Fed) Subject 6 [40]	CP021388	2377562	1916
7	*Bifidobacterium breve* NRBB18	Infant Isolate Subject 1 (Formula Fed) [40]	CP023193	2289686	1874
8	*Bifidobacterium breve* NRBB50	Infant isolate (Breast Fed) Subject 903 [40]	CP021391	2409058	2021
9	*Bifidobacterium breve* NRBB51	Infant isolate (Breast Fed) [40]	CP021392	2402272	1960
10	*Bifidobacterium breve* NRBB52	Infant isolate (Breast Fed), subject 903 [40]	CP021393	2379672	1989
11	*Bifidobacterium breve* NRBB56	Infant isolate, NIZO 658 [40]	CP021394	2425122	1985
12	*Bifidobacterium breve* CNCM I-4321	Child isolate CNCM I-4321 [40]	CP021559	2464852	2082
13	*Bifidobacterium breve* DRBB26	Child isolate [40]	CP021390	2396387	1978
14	*Bifidobacterium breve* DRBB27	Infant Isolate [40]	CP021552	2435083	2079
15	*Bifidobacterium breve* DRBB28	Infant Isolate [40]	CP021553	2462170	2096
16	*Bifidobacterium breve* 017W4-39	Infant Isolate [40]	CP021554	2301422	1923
17	*Bifidobacterium breve* 082W4-8	Infant isolate (Breast Fed) [40]	CP021555	2286339	1885
18	*Bifidobacterium breve* 180W8-3	Infant isolate [40]	CP021557	2273173	1891
19	*Bifidobacterium breve* 139W4-23	Infant isolate [40]	CP021556	2411276	2013
20	*Bifidobacterium breve* 215W4-47a	Infant isolate [40]	CP021558	2589602	2219

Table S2. Bifidobacterial strains used for computing the *B. breve* pan-genome.

	Organism/Name	Size (Mb)	GC%	Accession number/WGS	Status	Scaffolds
1	*Bifidobacterium breve* UCC2003	2.42268	58.7	CP000303.1	COMPLETE	1
2	*Bifidobacterium breve* ACS-071-V-Sch8b	2.32749	58.7	CP002743.1	COMPLETE	1
3	*Bifidobacterium breve* 12L	2.24462	58.9	CP006711.1	COMPLETE	1
4	*Bifidobacterium breve* JCM 7017	2.28892	58.7	CP006712.1	COMPLETE	1
5	*Bifidobacterium breve* JCM 7019	2.35901	58.6	CP006713.1	COMPLETE	1
6	*Bifidobacterium breve* NCFB 2258	2.3159	58.7	CP006714.1	COMPLETE	1
7	*Bifidobacterium breve* 689b	2.33171	58.7	CP006715.1	COMPLETE	1
8	*Bifidobacterium breve* S27	2.29446	58.7	CP006716.1	COMPLETE	1
9	*Bifidobacterium breve* BR3	2.42601	59.11	CP010413.1	COMPLETE	2
10	*Bifidobacterium breve* DSM20213	2.26941	58.9	AP012324.1	COMPLETE	1
11	*Bifidobacterium breve* NRBB01	2269404	58.8	CP021384	COMPLETE	1
12	*Bifidobacterium breve* NRBB02	2289884	58.5	CP021385	COMPLETE	1
13	*Bifidobacterium breve* NRBB04	2324647	58.7	CP021386	COMPLETE	1
14	*Bifidobacterium breve* NRBB08	2289759	58.5	CP023192	COMPLETE	1
15	*Bifidobacterium breve* NRBB09	2265557	58.6	CP021387	COMPLETE	1
16	*Bifidobacterium breve* NRBB11	2377562	58.7	CP021388	COMPLETE	1
17	*Bifidobacterium breve* NRBB18	2289686	58.5	CP023193	COMPLETE	1
18	*Bifidobacterium breve* NRBB19	2289726	58.5	CP023194	COMPLETE	1
19	*Bifidobacterium breve* NRBB20	2289892	58.5	CP023195	COMPLETE	1
20	*Bifidobacterium breve* NRBB27	2289838	58.5	CP023196	COMPLETE	1
21	*Bifidobacterium breve* NRBB49	2289791	58.5	CP023197	COMPLETE	1
22	*Bifidobacterium breve* NRBB50	2409058	58.8	CP021391	COMPLETE	1
23	*Bifidobacterium breve* NRBB51	2402272	59	CP021392	COMPLETE	1
24	*Bifidobacterium breve* NRBB52	2379672	58.8	CP021393	COMPLETE	1
25	*Bifidobacterium breve* NRBB56	2425122	58.9	CP021394	COMPLETE	1
26	*Bifidobacterium breve* NRBB57	2510381	59.3	CP021389	COMPLETE	1
27	*Bifidobacterium breve* CNCM I-4321	2464852	58.9	CP021559	COMPLETE	1
28	*Bifidobacterium breve* DRBB26	2396387	58.5	CP021390	COMPLETE	1

29	*Bifidobacterium breve* DRBB27	2435083	58.8	CP021552	COMPLETE	1
30	*Bifidobacterium breve* DRBB28	2462170	58.9	CP021553	COMPLETE	1
31	*Bifidobacterium breve* DRBB29	2435086	58.9	CP023198	COMPLETE	1
32	*Bifidobacterium breve* DRBB30	2471118	58.9	CP023199	COMPLETE	1
33	*Bifidobacterium breve* 017W4-39	2301422	58.7	CP021554	COMPLETE	1
34	*Bifidobacterium breve* 082W4-8	2286339	58.8	CP021555	COMPLETE	1
35	*Bifidobacterium breve* 180W8-3	2273173	58.8	CP021557	COMPLETE	1
36	*Bifidobacterium breve* 139W4-23	2411276	58.6	CP021556	COMPLETE	1
37	*Bifidobacterium breve* 215W4-47a	2589602	59.2	CP021558	COMPLETE	1
38	*Bifidobacterium breve* DSM20213	2.33139	58.5	ACCG02	DRAFT	103
39	*Bifidobacterium breve* HPH0326	2.50431	59.1	ATCB01	DRAFT	4
40	*Bifidobacterium breve* JCP7499	2.36972	58.6	AWSX01	DRAFT	68
41	*Bifidobacterium breve* GED8481	2.35844	58.6	LRPP01	DRAFT	29
42	*Bifidobacterium breve* DPC 6330	2.38623	58.6	AFXX01	DRAFT	47
43	*Bifidobacterium breve* CECT 7263	2.3144	58.9	AFVV01	DRAFT	34
44	*Bifidobacterium breve* 2L	2.24072	58.9	AWUG01	DRAFT	6
45	*Bifidobacterium breve* LMG 13208	2.26378	58.9	JGYR01	DRAFT	31
46	*Bifidobacterium breve* DSM20213	2.25713	58.9	JDUD01	DRAFT	29
47	*Bifidobacterium breve* BBRI4	2.42672	58.7	LFII01	DRAFT	28
48	*Bifidobacterium breve* MCC 0476	2.23381	58.6	AVQB01	DRAFT	15
49	*Bifidobacterium breve* MCC 1114	2.48728	59	AVQC01	DRAFT	33
50	*Bifidobacterium breve* MCC 1128	2.48022	58.9	AVQD01	DRAFT	25
51	*Bifidobacterium breve* MCC 0121	2.43634	58.9	AVQA01	DRAFT	27
52	*Bifidobacterium breve* MCC 1604	2.20639	58.7	AVQE01	DRAFT	15
53	*Bifidobacterium breve* BR-06	2.6608	59.2	BCXL01	DRAFT	101
54	*Bifidobacterium breve* BR-07	2.24948	58.7	BCXM01	DRAFT	27
55	*Bifidobacterium breve* BR-10	2.34992	58.8	BCXN01	DRAFT	60
56	*Bifidobacterium breve* BR-14	2.52987	59.2	BCXO01	DRAFT	57
57	*Bifidobacterium breve* BR-15	2.3738	58.7	BCXP01	DRAFT	27
58	*Bifidobacterium breve* BR-19	2.32499	58.6	BCXQ01	DRAFT	15

59	*Bifidobacterium breve* BR-20	2.33547	58.8	BCXR01	DRAFT	52
60	*Bifidobacterium breve* BR-21	2.61462	59.2	BCXS01	DRAFT	66
61	*Bifidobacterium breve* BR-A29	2.38763	58.8	BCXT01	DRAFT	62
62	*Bifidobacterium breve* BR-C29	2.28766	58.9	BCXU01	DRAFT	41
63	*Bifidobacterium breve* BR-H29	2.47084	59.2	BCXV01	DRAFT	39
64	*Bifidobacterium breve* BR-I29	2.24992	58.7	BCXW01	DRAFT	39
65	*Bifidobacterium breve* BR-L29	2.34466	59	BCXX01	DRAFT	44
66	*Bifidobacterium breve* RP2	2.26049	58.7	FNFW01	DRAFT	25
67	*Bifidobacterium breve* MCC 1605	2.32401	59	AWFV01	DRAFT	37
68	*Bifidobacterium breve* MCC 0305	2.28684	58.8	AWFR01	DRAFT	22
69	*Bifidobacterium breve* MCC 1094	2.32721	59	AWFS01	DRAFT	23
70	*Bifidobacterium breve* MCC 1340	2.37311	58.6	AWFT01	DRAFT	23
71	*Bifidobacterium breve* MCC 1454	2.45745	58.5	AWFU01	DRAFT	14
72	*Bifidobacterium breve* 31L	2.26565	58.6	AWUF01	DRAFT	4
73	*Bifidobacterium breve* JCM 1192	2.23281	58.7	BBAO01	DRAFT	143

Table S3. Substrates used for assessing growth capabilities in *B. breve*.

	Substrate	Type	Formula
1	2'-Fucosyllactose	Oligosaccharide	$C_{18}H_{32}O_{15}$
2	Adenosine	Nucleoside	$C_{10}H_{13}N_5O_4$
3	Amylopectin	Polysaccharide (plants)	$C_{30}H_{52}O_{26}$
4	Arabinose	Monosaccharide	$C_5H_{10}O_5$
5	Arabinoxylan	Polysaccharide (plants)	$C_{40}H_{64}O_{32}$
6	Arbutin	Glycosilated hydroquinone (plants)	$C_{12}H_{16}O_7$
7	β-cyclodextrin	Cyclic oligosaccharide (food additive)	$C_{42}H_{70}O_{35}$
8	Cellobiose	Disaccharide (plants)	$C_{12}H_{22}O_{11}$
9	Cellulose	Polysaccharide (plants)	$(C_6H_{10}O_5)_n$
10	Cyclomaltoheptaose	Cyclic oligosaccharide (food additive)	$C_{42}H_{72}O_{36}$
11	Cytidine	Nucleoside (Cytosine β-D-riboside)	$C_9H_{13}N_3O_5$
12	D-arabitol	Sugar alcohol	$C_5H_{12}O_5$
13	Deoxyadenosine	Deoxyribonucleoside	$C_{10}H_{13}N_5O_3$
14	Deoxy-D-ribose	Monosaccharide	$C_5H_{10}O_4$
15	Deoxy-L-ribose	Monosaccharide	$C_5H_{10}O_4$
16	D-lyxose	Monosaccharide	$C_5H_{10}O_5$
17	D-maltitol	Sugar alcohol	$C_{12}H_{24}O_{11}$
18	D-mandelonitrile-β-gentiobioside	Cyanogenic glycoside (plants)	$C_{20}H_{27}NO_{11}$
19	Deoxycitidine	Deoxyribonucleoside	$C_9H_{13}N_3O_4$
20	D-Salicin	Alcoholic β-glucoside (plants)	$C_{13}H_{18}O_7$
21	D-Turanose	Disaccharide (plants)	$C_{12}H_{22}O_{11}$
22	Esculin	Coumarin glucoside (plants)	$C_{15}H_{16}O_9$
23	Fructooligosaccharides	Oligosaccharide (plants)	-
24	Fructose	Monosaccharide	$C_6H_{12}O_6$
25	Fucose	Monosaccharide	$C_6H_{12}O_5$
26	Galactan	Polysaccharide (plants)	$C_{20}H_{36}O_{16}$
27	Galactose	Monosaccharide	$C_6H_{12}O_6$
28	Galacturonic acid	Sugar acid	$C_6H_{10}O_7$
29	γ-cyclodextrin	Cyclic oligosaccharide (food additive)	$C_{48}H_{80}O_{40}$
30	Glucan	Polysaccharides	$(C_6H_{10}O_5)_n$
31	Glucose	Monosaccharide	$C_6H_{12}O_6$
32	Glucuronic acid	Sugar acid	$C_6H_{10}O_7$
33	Glycogen	Polysaccharide	$(C_6H_{10}O_5)_n$
34	Galactooligosaccharides	Oligosaccharide	-
35	Inosine	Nucleoside	$C_{10}H_{12}N_4O_5$
36	Inulin	Polysaccharides (plants)	$C_{6n}H_{(10n+2)}O_{(5n+1)}$
37	Lactitol	Sugar alcohol	$C_{12}H_{24}O_{11}$

38	Lactobionic acid	Sugar acid	$C_{12}H_{22}O_{12}$
39	Lactose	Disaccharide	$C_{12}H_{22}O_{11}$
40	Lactulose	Disaccharide	$C_{12}H_{22}O_{11}$
41	L-arabitol	Sugar alcohol	$C_5H_{12}O_5$
42	Lacto-*N*-neotetraose	Oligosaccharide (human milk)	$C_{26}H_{45}NO_{21}$
43	Maltodextrin	Polysaccharide (food additive)	$C_{6n}H_{(10n+2)}O_{(5n+1)}$
44	Maltose	Disacharide	$C_{12}H_{22}O_{11}$
45	Mannitol	Sugar alcohol	$C_6H_{14}O_6$
46	Mannose	Monosaccharide	$C_6H_{12}O_6$
47	Melezitose	Trisaccharide (plants)	$C_{18}H_{32}O_{16}$
48	Melibiose	Disaccharide	$C_{12}H_{22}O_{11}$
49	Methyl-D-galactopyranoside	Monosaccharide	$C_7H_{14}O_6$
50	Methyl-D-glucopyranoside	Monosaccharide	$C_7H_{14}O_6$
51	Mucin	Glycan (host derived)	-
52	Myo-inositol	Sugar alcohol	$C_6H_{12}O_6$
53	*N*-acetylgalactosamine	Amino sugar	$C_8H_{15}NO_6$
54	*N*-acetylmannosamine	Amino sugar	$C_8H_{15}NO_6$
55	*N*-acetylglucosamine	Amino sugar	$C_8H_{15}NO_6$
56	Palatinose	Disaccharide	$C_{12}H_{22}O_{11}$
57	Pectin	Polysaccharide (plants)	$C_6H_{10}O_7$
58	Pectin acidic	Polysaccharide (plants)	$C_6H_{10}O_7$
59	Psicose	Monosaccharide	$C_6H_{12}O_6$
60	Pullulan	Polysaccharide	$(C_6H_{12}O_5)_n$
61	Raffinose	Trisaccharide (plants)	$C_{18}H_{32}O_{16}$
62	Raftiline	Polysaccharides (plants)	$C_{6n}H_{(10n+2)}O_{(5n+1)}$
63	Rhamnose	Monosaccharide	$C_6H_{12}O_5$
64	Ribose	Monosaccharide	$C_5H_{10}O_5$
65	Saponin	Glycosides (plants)	$C_{27}H_{42}O_3$
66	Sialic acid	Glycan (host derived)	$C_{11}H_{19}NO_9$
67	Sorbitol	Sugar alcohol	$C_6H_{14}O_6$
68	Sorbose	Monosaccharide ketose	$C_6H_{12}O_6$
69	Starch	Polysaccharide (plants)	$(C_6H_{10}O_5)_n$
70	Sucralose	Sugar chlorinate	$C_{12}H_{19}Cl_3O_8$
71	Sucrose	Disaccharide	$C_{12}H_{22}O_{11}$
72	Tagatose	Monosaccharide	$C_6H_{12}O_6$
73	Thymidine	Deoxynucleoside	$C_{10}H_{14}N_2O_5$
74	Uridine	Glycosilated pyrimidine analog	$C_9H_{12}N_2O_6$
75	Xylitol	Sugar alcohol	$C_5H_{12}O_5$
76	Xylooligosaccharides	Oligosaccharide (plants)	-
77	Xylose	Monosaccharide (plants)	$C_5H_{10}O_5$

Table S4. Antimicrobials used in this study.

Antibiotic	Class	Purpose
Tetracycline	Tetracycline	Initial screening
Erythromycin	Macrolide	Initial screening/GTM assessment
Streptomycin	Aminoglycoside	Initial screening/ GTM assessment
Kanamycin	Aminoglycoside	Initial screening/GTM assessment
Neomycin	Aminoglycoside	Initial screening
Gentamicin	Aminoglycoside	Initial screening
Chloramphenicol	Chloramphenicol	Initial screening
Amikacin	Aminoglycoside	GTM assessment
Tobramycin	Aminoglycoside	GTM assessment
Bleomycin	Aminoglycoside	GTM assessment
Netilmicin	Aminoglycoside	GTM assessment

Table S5. Oligonucleotide primers used in this study.

Purpose	Primer	Sequence*
Cloning of Bbr_0020 gene	0020F	cattac**aagctt**ccacaacctctgatttcgtgc
	0020R	ggttaa**tctaga**cgtccagttcttgaggtctttag
Confirmation of site-specific homologous recombination (Bbr_0020)	0020conf	gctgtactaccaattccctgg
	TetWF	tcagctgtcgacatgctcatgtacggtaag
Cloning of Bbr_0021 gene	0021F	ggttac**aagctt**cgattactacgcagacatgtcc
	0021R	ggttaa**tctaga**gaagttgcgtcacaccttgatcg
Confirmation of site-specific homologous recombination (Bbr_0021)	0021conf	cgtctgacacatacgaacag
	TetWF	tcagctgtcgacatgctcatgtacggtaag
Cloning of NRBB51_1114 gene	1114F	ttcgca**ctgcag**gggcggctatttgtcatg
	1114R	cgtccg**aagctt**cttgtgccacgcccgaac
Cloning of CNCMI4321_0985-87 locus	0985-87F	tagcat**ctgcag**atggcggatgaaatgcgac
	0985-87R	gcatct**aagctt**agcggcgacgcatccaaatc
Cloning of CNCMI4321_0985 gene	0985F	tagcat**ctgcag**attagcttcttggggtatc
	0985R	gcatct**aagctt**agcggcgacgcatccaaatc
Cloning of CNCMI4321_0986 gene	0986F	tagcat**ctgcag**ggtaaaaccggctgcctggatagc
	0986R	gcatct**aagctt**gacgaactccaattcactg
Cloning of CNCMI4321_0987 gene	0987F	tagcat**ctgcag**atggcggatgaaatgcgac
	0987R	gcatct**aagctt**cacctcaaatggttcgctg

* Restriction sites indicated in bold

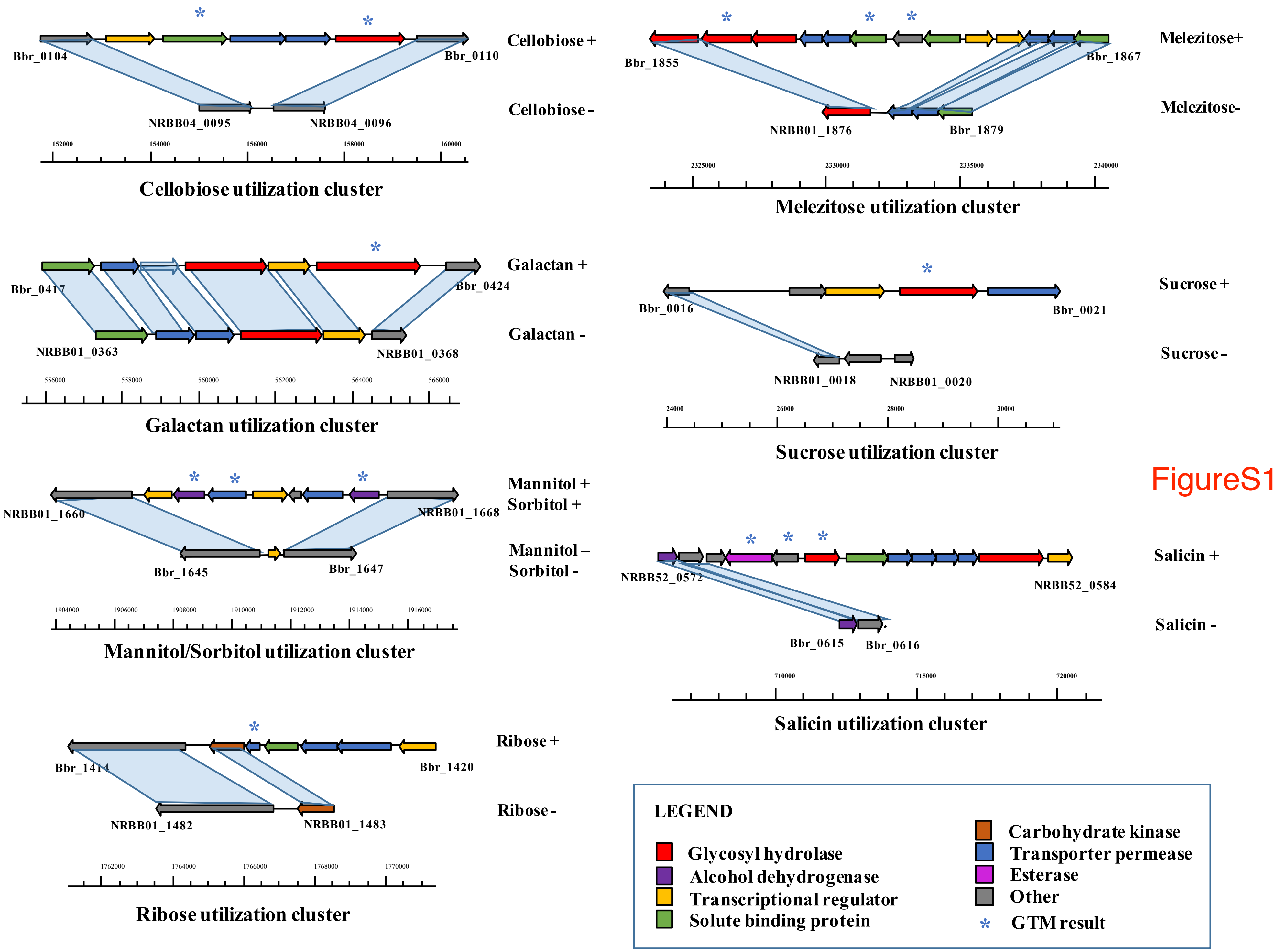
Bbr_0104
Bbr_0110
Cellobiose +
Cellobiose -
NRBB04_0095
NRBB04_0096
152000
154000
156000
158000
160000
Cellobiose utilization cluster
Bbr_1855
Bbr_1867
Melezitose+
Melezitose-
NRBB01_1876
Bbr_1879
2325000
2330000
2335000
2340000
Melezitose utilization cluster
Bbr_0417
Bbr_0424
Galactan +
Galactan -
NRBB01_0363
NRBB01_0368
556000
558000
560000
562000
564000
566000
Galactan utilization cluster
Bbr_0016
Bbr_0021
Sucrose +
Sucrose -
NRBB01_0018
NRBB01_0020
24000
26000
28000
30000
Sucrose utilization cluster
NRBB01_1660
NRBB01_1668
Mannitol +
Sorbitol +
Mannitol –
Sorbitol -
Bbr_1645
Bbr_1647
1904000
1906000
1908000
1910000
1912000
1914000
1916000
Mannitol/Sorbitol utilization cluster
NRBB52_0572
NRBB52_0584
Salicin +
Salicin -
Bbr_0615
Bbr_0616
710000
715000
720000
Salicin utilization cluster
Bbr_1414
Bbr_1420
Ribose +
Ribose -
NRBB01_1482
NRBB01_1483
1762000
1764000
1766000
1768000
1770000
Ribose utilization cluster
LEGEND
Glycosyl hydrolase
Alcohol dehydrogenase
Transcriptional regulator
Solute binding protein
Carbohydrate kinase
Transporter permease
Esterase
Other
GTM result

FigureS1

FigureS2

Exopolysaccharide biosynthesis locus in *B. breve*

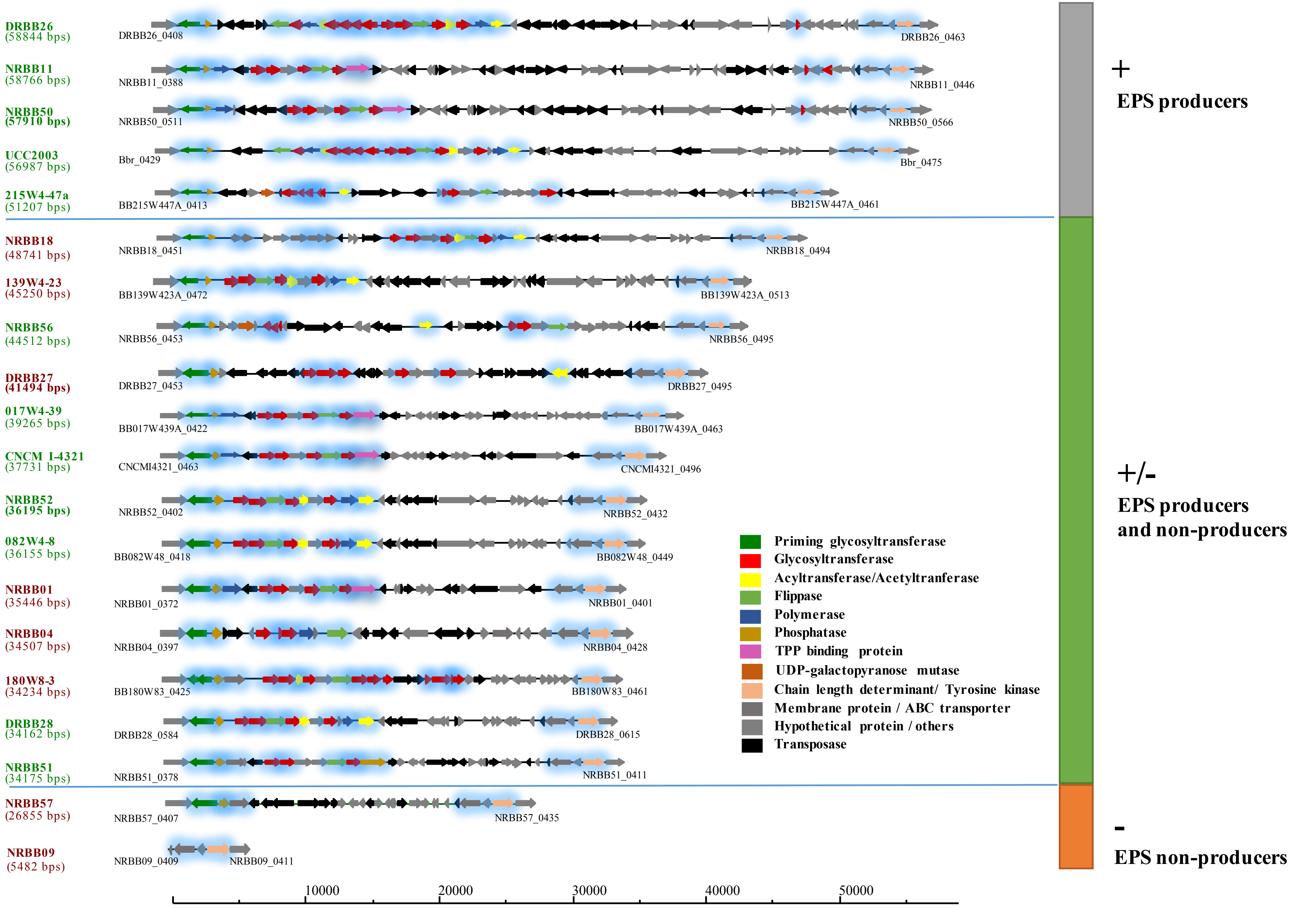

Supplement: Supplementary file 1 — Supplementary material [file 41598_2018_28919_MOESM1_ESM.pdf]
